# Neural Correlates of Social Exclusion and Childhood Trauma in Borderline Personality Disorder

**DOI:** 10.7759/cureus.92738

**Published:** 2025-09-19

**Authors:** Mohammad Asif Khan, Rinky Sharma

**Affiliations:** 1 Primary and Mental Health, Base Care Foundation, London, GBR; 2 General Medicine, Luton & Dunstable University Hospital, Luton, GBR

**Keywords:** borderline personality disorder, childhood trauma, fmri, functional magnetic resonance imaging, neural correlates, personality disorder, social exclusion

## Abstract

Introduction

Borderline personality disorder (BPD) is a cluster B personality disorder that is characterized by altered perceptions of self-image, affect, and behavioral difficulties, especially a fear of being abandoned or rejected. Individuals with BPD engage in impulsive behaviors such as substance abuse, risky sexual behavior, self-harm, excessive spending, or binge eating. Beyond the individual level, BPD has a damaging effect on the integrity and safety of societies. Subjects with borderline personality disorder have various types of traumatic childhood experiences and social exclusion. Evaluating neural substrates of social exclusion and childhood trauma in BPD using functional magnetic resonance imaging (fMRI) can be a reliable approach to assess the regions of the brain that are affected in response to social exclusion and childhood maltreatment.

Methods

The study used secondary data obtained from OpenNeuro. In this study, fMRI was used to acquire task-based blood-oxygen-level-dependent (BOLD) signals in the participants' brains while they performed the Cyberball social exclusion task. Subjects were playing a virtual game of tossing with two other players. We conducted the fMRI analysis to compare voxel-wise activations in response to social exclusion while experimentally manipulating the percentages of participation (0%, 33%, 66%, and 100%) in the game with 20 patients with BPD and 16 age- and sex-matched controls. The study was aimed at determining the neural correlates of social exclusion and childhood trauma in individuals with BPD.

Results

The fMRI analysis revealed higher signalchanges in areas including anterior insula (AIns), middle frontal gyrus (MFG),cuneus (Cun), posterior orbital gyrus (POrG), medial orbital gyrus (MOrG),inferior temporal gyrus (ITG), fusiform gyrus (FuG), and postcentral gyrus(PoG) related to the effect of childhood trauma. Further analysis for neuralunderpinnings of the expression of social exclusion in individuals with BPDdetected areas including AIns, superior frontal gyrus (SFG), MFG, precentralgyrus (PrG), supramarginal gyrus (SMG), superior parietal lobule (SPL),inferior occipital gyrus (IOG), FuG, lingual gyrus (LiG), calcarine cortex(Calc), and Cun.

Conclusion

Our voxel-based analysis of the neural underpinnings of social exclusion expression detected larger signal changes for exclusion compared to inclusion in voxels involved in emotional regulation, suggesting an increased sensitivity to exclusion or a lower level of inclusion, possibly explained by the anticipation of abandonment in individuals with BPD. Hence, individuals with BPD may exhibit readiness to perceive and overreact to social abandonment, affecting their social and psychological adjustments.

## Introduction

Borderline personality disorder (BPD) is one of four cluster B personality disorders, including borderline, antisocial, narcissistic, and histrionic. BPD is an incapacitating psychiatric illness characterized by altered perceptions of self-image, affect, and behavioral difficulties, especially a fear of being abandoned or rejected. The fourth edition of the Diagnostic and Statistical Manual of Mental Disorders (DSM-IV) contains the code for BPD as 301.83. The criteria include a pervasive pattern of instability in interpersonal relationships, self-image, and affect, with marked impulsivity, beginning in early adulthood and present in a variety of contexts, as indicated by five (or more) of the following: 1) frantic efforts to avoid real or imagined abandonment; 2) unstable and intense relationships: characterized by alternating between extremes of idealization and devaluation (the “splitting” pattern); 3) identity disturbance: markedly unstable self-image or sense of self; 4) impulsivity in at least two areas that are potentially self-damaging: e.g., spending, sex, substance abuse, reckless driving, binge eating; 5) recurrent suicidal behavior, gestures, threats, or self-mutilating behavior; 6) affective instability: marked reactivity of mood (e.g., intense episodic dysphoria, irritability, anxiety, usually lasting a few hours, rarely more than a few days); 7) chronic feelings of emptiness; 8) inappropriate, intense anger or difficulty controlling anger: e.g., frequent temper outbursts, constant anger, recurrent physical fights; 9) transient, stress-related paranoid ideation or severe dissociative symptoms [1].

Growing interest has lately been shown in the neurological underpinnings of these social symptoms and how they relate to experiences in childhood [2]. BPD causes significant impairment and distress and is associated with multiple medical and psychiatric comorbidities. Surveys have estimated the prevalence of BPD to be 1.6% in the general population, 10% to 12% in outpatient psychiatric clinics, and 20% to 22% in inpatient clinics [3].

The psychopathology of BPD is related to disturbed emotion processing and emotion dysregulation, cognitive disturbances including dissociation, behavioral dysregulation and impulsivity, interpersonal disturbances, and altered pain perception [4]. The main characteristics of BPD include instability in interpersonal relationships, self-image, and mood, mixed with severe impulsivity, commencing in early adulthood: unstable self-image; impulsivity in multiple self-destructive ways (e.g., spending, sex, substance abuse, binge eating, reckless driving) [5]. Patients with BPD also have an increased suicidal risk due to a significant association with lifetime suicidal ideation, last-year suicidal ideation, suicide plans, and suicide attempts [6].

Subjects with BPD have a strong association with childhood abuse compared with those without BPD [7], and childhood maltreatment (physical, sexual, or neglect) is found in the former, hinting at an association between BPD and childhood maltreatment and suggesting a link between the development of BPD and the severity of traumatic exposure, as shown by earlier onset of trauma, trauma of an assaultive and personal nature, and exposure to different types of traumatic events. 

In the context of the executive and cognitive functions of the brain for an individual, the Default Mode Network (DMN) is a large-scale brain network that shows high activity during rest and self-referential thinking, such as daydreaming, recalling memories, or imagining the future, and decreased activity during externally focused tasks. It primarily involves the medial prefrontal cortex (PFC), posterior cingulate cortex/precuneus (PCu), and angular gyrus, and is thought to play a key role in self-awareness, social cognition, and internal mentation. In contrast, the fronto-limbic circuit connects prefrontal cortical regions (especially the ventromedial and dorsolateral PFC) with limbic structures such as the amygdala, hippocampus, and anterior cingulate cortex (ACC), and is critical for emotional regulation, impulse control, and stress response. Dysregulation in these networks has been implicated in psychiatric disorders including BPD, depression, and anxiety, with DMN abnormalities linked to disturbed self-representation and fronto-limbic dysfunction contributing to affective instability and poor emotion regulation [8].

In individuals with BPD, childhood trauma experiences have a detrimental effect on regions involved in cognitive and executive functions exerted by these DMN and fronto-limbic circuit, such as the temporal lobe, insular cortex, and parahippocampal gyrus (PHG). Childhood abuse is linked with functional reorganizations of regions for damaged emotion representation, and the altered communications in the temporal cortex and supplementary motor cortex are related to emotional and physical abuse [8]. Additionally, in the superior temporal cortex (STC) and middle ACC, decreased inclusion responses are observed in association with higher levels of childhood maltreatment [2]. Patients with BPD exposed to higher levels of childhood trauma (i.e., physical abuse) also show decreased activation in the right cuneus (Cun) and increased neuronal signals in the midbrain, pulvinar, and middle frontal gyrus (MFG) in response to negative emotions [9].

On a further note, stressful life events and traumatic experiences, such as persistent sexual or physical abuse or emotional or physical neglect, alter the function of the limbic system and hypothalamus-pituitary-adrenal (HPA) axis, particularly the hippocampus, which plays a significant role in the cognitive processes involved in learning and long-term memory [10]. The fronto-limbic network includes regions involved in emotional processing like the amygdala and insula, and the frontal brain regions engaged in regulatory control processes like ACC, MFG, orbitofrontal cortex (OFC), and dorsolateral prefrontal cortex (DL-PFC) are implicated in individuals with BPD, demonstrated by structural and functional changes in these networks. There is a probable connection between disrupted emotion processing and other prominent features of BPD, such as impulsivity and interpersonal difficulties due to limbic hyperreactivity and decreased recruitment of the frontal brain regions [11].

Large-scale population data from Swedish national registries (over 1.8 million individuals born between 1973 and 1993, with 11,665 diagnosed cases of BPD show clear evidence of familial aggregation and genetic contribution to BPD. The risk of diagnosis decreased with genetic distance. It was highest in monozygotic twins (hazard ratio or HR of 11.5, followed by dizygotic twins (HR 7.4), full siblings (HR 4.7), and lower in half-siblings and cousins. Using structural equation modelling, the heritability of BPD was estimated at 46% (95% CI 39-53), with the remaining variance explained largely by unique environmental factors rather than shared family environment. These findings confirm a moderate genetic propensity for BPD but also highlight the crucial role of individual environmental exposures and gene-environment interplay in the disorder’s development [12]. However, BPD arises from a complex interplay of genetic vulnerability and environmental stressors, not one single cause. Heritability is significant, but early life trauma and unstable attachments are often the “triggers” that shape expression of the disorder [4].

The efforts to understand the neurobiological origins in conjunction with the social determinants of BPD have rapidly increased, and over the last decades, neuroimaging has become one of the most important methods to investigate neurobiological alterations possibly underlying core neurobiological features of BPD [13]. The neuroimaging techniques have become one of the most frequent approaches to spot abnormalities in BPD patients to detect structural and volumetric differences in various brain regions using techniques like structural MRI (sMRI), functional MRI (fMRI), and diffusion tensor imaging (DTI), and the domains of fMRI findings are focused on the detection of affective dysregulation, interactions of detachment, self-harming behavior, and pain processing, and social interaction [14].

Out of different neuroimaging modalities, fMRI uses strong magnetic fields and radio waves (like MRI) detected by an MRI scanner, which measures changes in blood oxygenation level-dependent (BOLD) signals (when a region of the brain is more active, it consumes more oxygen, and local blood flow increases). The machine detects these changes and produces real-time images of brain activity. fMRI is commonly used for diverse research applications, including neuroscience research to study brain functions (language, memory, motor control, decision-making), understanding emotions, behavior, and learning; research in psychiatric disorders (e.g., depression, schizophrenia, dementia); pre-surgical brain mapping (e.g., localizing speech/motor areas before removing a brain tumor); and assessing functional brain recovery after stroke. Benefits of fMRI include non-invasive (no radiation, unlike positron emission tomography (PET)/CT) brain mapping, high spatial resolution (pinpoints active brain regions), can show real-time brain activity, and ensures repeated safety (no cumulative radiation risk).

In recent times, fMRI has provided increasing insight into the neural correlates of BPD. Recent meta-analyses on disturbed emotion processing in BPD reported a consistent pattern of altered function and structure in the cortical regions, including the PFC and temporal cortex, along with limbic regions, i.e., the amygdala and insula [15]. Interestingly, an fMRI-based evaluation can even evaluate non-pharmacological interventions like dialectical behavior therapy (DBT), which is frequently used to treat patients with BPD, and anticipate how well they will work. Using functional and structural MRI data of anatomical regions multimodally impacted in BPD, such as the amygdala and parahippocampal activation during a cognitive reappraisal task, as well as the amygdala's grey matter volume, provides the strongest prediction for a DBT response [16].

In this study, we aimed to assess the regions of the brain that get activated in response to social exclusion using the Cyberball task/game, reflective of an environment of social abandonment, and effective in detecting emotional dysregulation due to childhood maltreatment [17]. In response to social exclusion, patients with BPD experience higher anticipation of social exclusion due to an altered perception of being more excluded from the game than the controls, who were influenced by their reduced level of inclusion in the game [18]. The Cyberball game is used in other fMRI studies comprising patients with BPD to detect activations involved in social brain processing and emotional dysregulation [19].

We adopted the Cyberball task to explore voxel-wise activations in response to inclusion and exclusion, while experimentally manipulating the percentages of participation in the game. For fMRI analysis, the fMRI data during the Cyberball task were obtained from OpenNeuro [20].The objective of the study was to compare the areas of brain activation in patients with BPD to those of healthy controls matched by age and sex. It sought to assess the differences in activations in response to social exclusion and childhood adversities, with an attempt to determine the brain areas affected by childhood trauma and social exclusion in individuals with BPD. We hypothesized that in patients with BPD, there would be larger activations for different levels of exclusion from the game compared to the controls in areas including hippocampus (HPC), OFC, ACC, amygdala, insula (Ins), MFG, PFC, middle temporal gyrus (MTG), and temporoparietal area concerning the effects of social exclusion, and MFG, MTG, insular cortex, PHG, STC, occipital cortex, and Cun linked to functional alteration of regions for damaged emotion due to childhood abuse in BPDs.

## Materials and methods

Participants

The data on the participants were obtained from an open source, "OpenNeuro Dataset ds000214," available on "OpenNeuro" [20]. The experiment consisted of the Cyberball social exclusion task. Twenty individuals hailing from Edinburgh, Scotland, were included in the study, fulfilling the diagnostic criteria for BPD using the Structured Clinical Interview for DSM-IV (SCID-II) [20], and were recruited from outpatient and support services around Edinburgh, Scotland. Twenty age- and sex-matched controls were also included among the healthy volunteers. Due to technical issues during the scanning, four controls were excluded from the study. All participants who were pregnant, not fit for MRI, with a history of head injury, existing substance dependence, or diagnosed with a psychotic disorder were excluded from the study. All participants provided written informed consent prior to inclusion in the study as per the ethical approval given by the Lothian National Health Service Research Ethics Committee. All participants were assessed using the Childhood TraumaQuestionnaire (CTQ) to evaluate the extent of the childhood trauma.

Experimental task

Participants performed the ‘Cyberball social exclusion task’ in ‘OpenNeuro’ [20] during fMRI to acquire task-based BOLD signals in the brain. Subjects were systematically included or excluded from the game while playing a virtual game of tossing balls with two other players to assess the neural responses to social exclusion in the form of different levels of exclusion from the game. The task was experimentally manipulated to provide corresponding levels of exclusion as 0%, 33%, 66%, and 100%, respectively, ensured by zero, one, two, and three throws to the participants. When the participants received the ball, they indicated which computer player they wished to throw it to with a button press. Each inclusion level was repeated four times, resulting in a total of 16 experimental blocks (mean duration of 24 seconds). The first block was 100% inclusion, and all following blocks were random and interleaved with 13-second rest blocks. 100% inclusion means that the participant received three throws for every nine-throw block, making their inclusion level equal to that of the other two players.

Modelling of the experimental task

We considered a mixed experimental design to ensure time-sensitive neuronal responses and sustained activity related to task-level processing, containing blocks of 100%, 66%, and 33% involvement in the game, and each block contained events of inclusion and exclusion, where the blocks were changing more slowly while the events were changing faster. 

fMRI image acquisitions

A 3T Siemens Magnetom Verio scanner (Seimens Healthineers, Erlangen, Germany) used for neuroimaging scanning at the Clinical Research Imaging Centre in Edinburgh. Axial acquisition of BOLD images by echo planar imaging (EPI) was obtained as 26 interleaved slices and 347 volumes at 3.4 x 3.4 x 5 mm resolution, while the in-plane resolution was 64 x 64. BOLD imaging was performed with a flip angle of 66°, a repetition time (TR) of 1.56 seconds, and a time to echo (TE) of 26 ms. For structural imaging, high-resolution T1 Magnetization-Prepared Rapid Gradient Echo (MPRAGE) was acquired using the parameters including 160 interleaved slices, resolution 1 x 1 x 1 mm, in-plane resolution 256 x 256 with flip angle 90°, TR 2300 ms, TE 2.98 ms, and field of view 256 x 256 mm.

fMRI image preprocessing 

Preprocessing of fMRI images from all subjects was done following the standard steps of motion correction, slice timing correction in an interleaved order, coregistration, normalization, smoothing using Statistical Parametric Mapping (SPM12) within MATLAB-R2022b (9.13, MathWorks, Massachusetts, USA), and finally averaging normalized T1 anatomical scans across the subjects in the order of the subject number for checking the final alignment. An additional step with preprocessing was done using the artefact detection tool (ART-2015) to characterize motion for comprehensive analysis of sources of artefacts in time-series data, including spiking and motion based on normalized functional scans and motion parameter files for a specific session for each subject. ART specified the high motion volumes in the data and excluded the outlier volumes from the analysis. For detecting the subjects with a larger extent of motion, ART provided graphs for average signal intensity over time, standard deviations of global mean, and volume-to-volume motion (compound motion combining three translations and rotations). We specified a threshold to determine the outliers as 2.5 mm based on half the maximum and the average signal intensity over time to detect the outlier volumes. For this study, we considered 15% of the volumes as the cut-off for detecting the outliers. The evaluation of ART files depicted that the number of spikes was less than the threshold for all the subjects; hence, we took forward all the subjects for the first-level analysis.

First-level analysis

We considered a commonly used method for reporting fMRI analysis, that is, an event-related factorial generalized linear model (GLM) model [21] for reporting the findings for every participant. For each subject, the factors were engagement (either inclusion or exclusion) and the level of involvement (100%, 66%, and 33%), making it into a 3×2 factorial design. The design matrix included columns for inclusion and exclusion for each of the percentage blocks, including 0%; each of the outliers in separate columns based on the estimation derived from ART analysis; three consecutive columns for rotations, three columns for translations; and one compound motion column. To detect the responses to the experimental task, we computed F-contrasts to examine the main effect of model inclusion vs. exclusion, 100% inclusion vs. 66% inclusion, 100% inclusion vs. 33% inclusion, and 66% inclusion vs. 33% inclusion, and the interaction effect of model inclusion × percentage (100×66), (100×33), and (66×33), at a significance level of p<0.001 at the voxel level for the group differences and significant voxels corresponding to family-wise error (FWE) correction of p<0.05 on the peak level for the group averages. The time of onset of specific events for each condition was derived from the dataset, and the durations were set to ‘0’ for all conditions, since it was considered an event-related experiment. First-level models were specified, altering the high-pass filter (HPF) to 180 from 128.

Second-level analysis

Specifications of models for the between-subjects analysis was done to compare the changes in BOLD signals in response to the task processing varied depending on the models: Inclusion vs. Exclusion, 100% Inclusion vs. 66% Inclusion, and 66% Inclusion vs. 33% Inclusion to see the main effects, and Inclusion × Percentage (100×66), (100×33), and (66×33) for the interaction effects. The comparison made between the controls and the patients with BPD was based on an F-contrast of group differences and group averages.

fMRI analysis

The fMRI analysis was conducted to detect voxels using F-contrasts for different models in a factorial experimental design with multiple levels [21], as specified for this study. We considered models to see the differences in main and interaction effects based on inclusion or exclusion and the extent of inclusion in the game in terms of percentages (0%, 33%, 66%, and 100%) manipulated experimentally. We computed the between-group comparisons based on group differences and group averages at peak-level family-wise error (pFWE) correction of p<0.05 (cluster-forming p<0.001), which is the most common approach for an analysis at the peaks of activation for whole-brain voxel-wise analysis [21]. Regions achieving a pFWE-corrected significance of p<0.05 in addition to the voxel-wise threshold of p<0.001 were reported for fMRI analyses.

## Results

Demographics 

The range of ages for the controls was 20 to 50 years, and for the patients with BPD, it was 21 to 51 years. Both groups had three male participants, with one left-handed individual in each group.

Main effects

**Figure 1:**
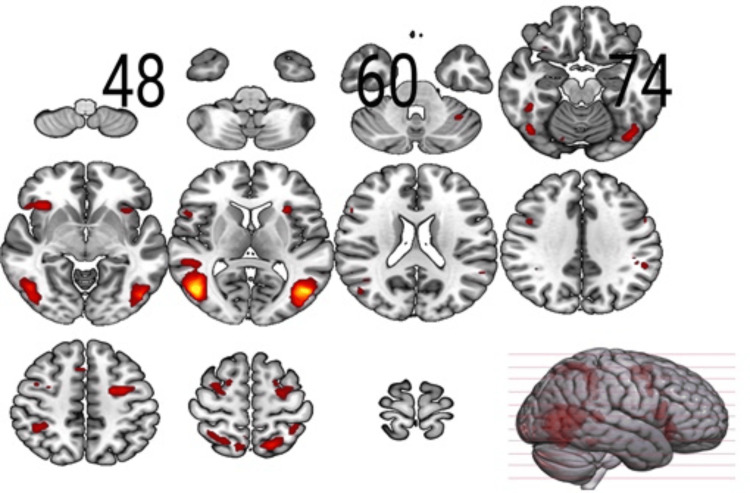
Sections of the brain showing the main effect (Group Average: Inclusion vs. Exclusion) Results for the F-contrast threshold at pFWE<0.05 (cluster-forming p<0.001); pFWE: peak-level family-wise error.

**Figure 2:**
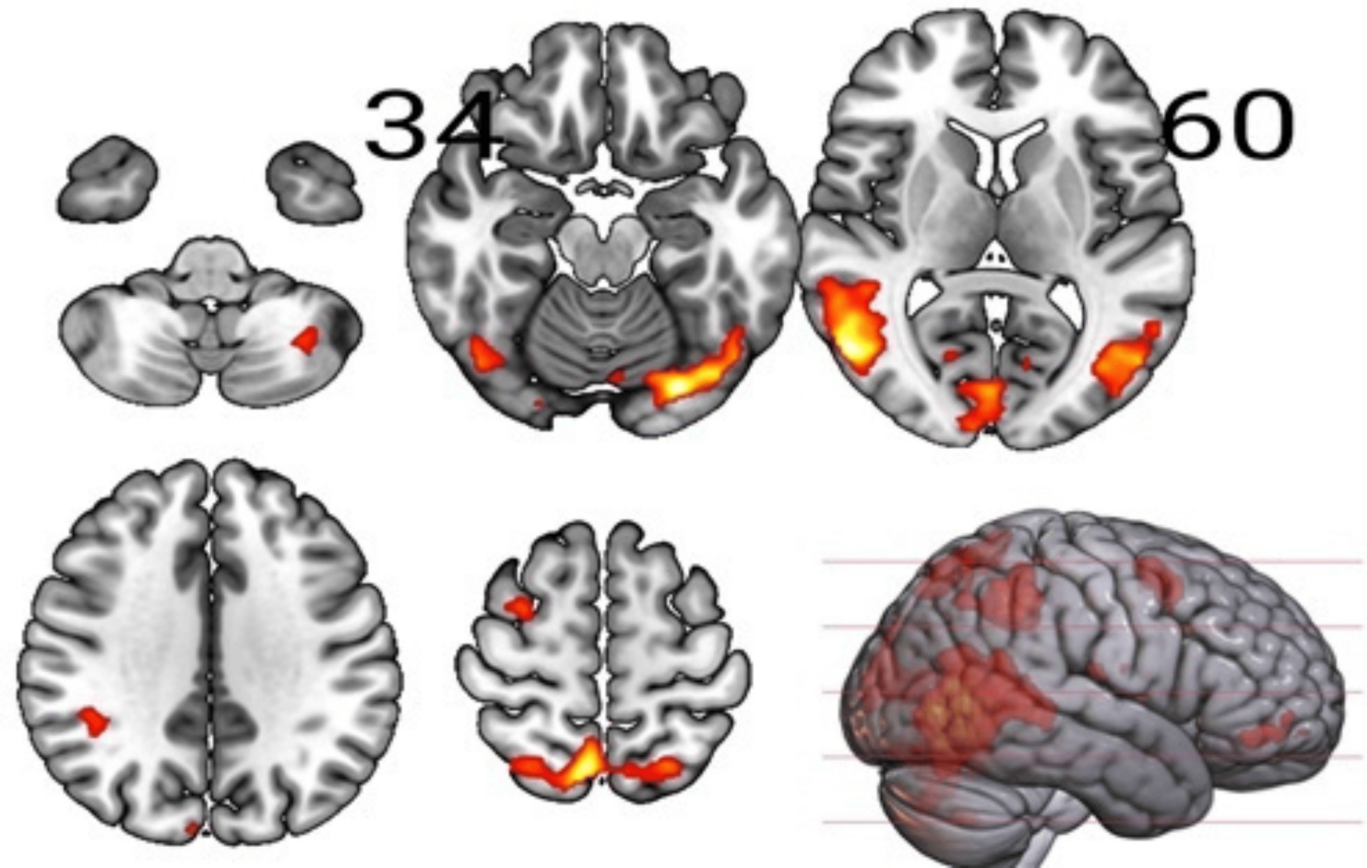
Sections of the brain showing the interaction effects, Inclusion x Percentage (100,66) Results for the F-contrast threshold at pFWE<0.05 (cluster-forming p<0.001); pFWE: peak-level family-wise error.

**Figure 3:**
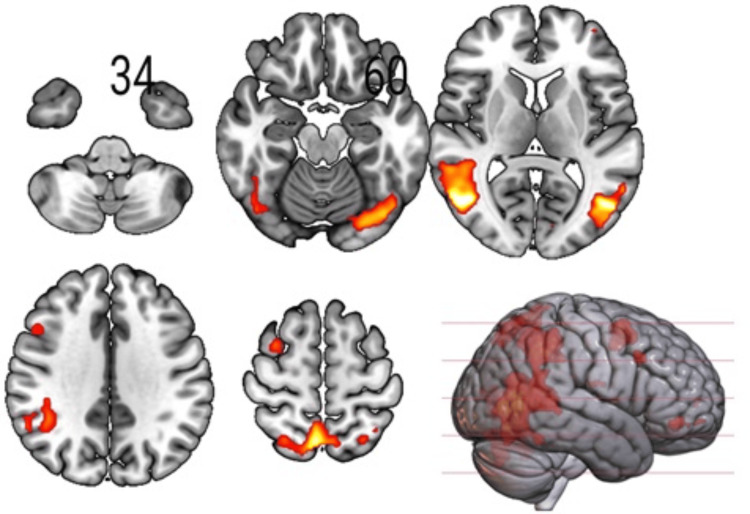
Sections of the brain showing the interaction effects, Inclusion x Percentage (100,33) Results for the F-contrast threshold at pFWE<0.05 (cluster-forming p<0.001); pFWE: peak-level family-wise error.

For the group average-based evaluation at pFWE<0.05 (cluster-forming p<0.001), there were cluster locations surviving for the models: Inclusion vs. Exclusion, as shown in Table 1 and Figure 1, 100% Inclusion vs. 66% Inclusion, as shown in Table 1 and Figure 2, and 100% Inclusion vs. 33% Inclusion, as shown in Table 1 and Figure 3.

**Table 1:** Cluster locations for the main effects (F-contrast) ^1^Locations based on the two largest intersections for the peak level correction at pFWE<0.05 (cluster forming p<0.001) based on the SPM neuromorphometrics atlas; pFWE: peak-level family-wise error; SPM: Statistical Parametric Mapping.

Inclusion vs Exclusion						
Cluster index	Cluster size	Peak F-statistic	Peak coordinates (mm)			Cluster location^1^
			x	y	z	
1	1776	196.5	46	-66	6	Inferior occipital gyrus (39.1%), White Matter (36.9%)
2	1411	159.8	-44	-68	6	White Matter (42.8%), Inferior occipital gyrus (27.9%)
3	495	59.95	38	-42	40	White Matter (54.8%), Superior parietal lobule (21.7%)
4	459	80.78	-22	-4	52	White Matter (47.6%), Superior frontal gyrus (24.1%)
5	346	78.22	32	2	56	Middle frontal gyrus (53.7%), Precentral gyrus (18.4%)
6	240	67.58	32	22	-10	White Matter (42.1%), Anterior insula (33.7%)
7	192	66.3	-30	20	4	White Matter (43.2%), Anterior insula (30.7%)
8	177	52.59	-50	-42	26	White Matter (53.1%), Supramarginal gyrus (19.2%)
9	38	59.48	42	-40	-20	Fusiform gyrus—FuG (50.5%), White Matter (28.5%)
10	19	41.55	4	-84	-2	Right lingual gyrus—LiG (29.3%), Left lingual gyrus—LiG (20.8%)
100% Inclusion vs. 66% Inclusion						
1	1	33.57	6	-72	12	Calcarine cortex- Calc (37.6%), Cuneus- Cun (17.5%)
100% Inclusion vs. 33% Inclusion						
1	14	36.8	-8	-74	2	Lingual gyrus—LiG (35.6%), Calcarine cortex—Calc (31.8%)

Based on the neuromorphometrics atlas from SPM12, we found significant (p<0.05 FEW-corrected at peak level) areas intersecting with the white matter regions as well as areas for main effects, including the anterior insula (AIns), superior frontal gyrus (SFG), MFG, precentral gyrus (PrG), supramarginal gyrus (SMG), superior parietal lobule (SPL), inferior occipital gyrus (IOG), fusiform gyrus (FuG), lingual gyrus (LiG), calcarine cortex (Calc), and Cun (Table 1 and Figure 1). Apart from the voxel-based analysis, the voxel-specific signal changes detected larger signal changes for the exclusion in comparison to the changes for inclusion, i.e., in SPL, MFG, IOG, and AIns, suggesting an increased sensitivity to exclusion due to the increased fear of abandonment in patients with BPD. 

Interaction effects

We also determined the interaction effects based on the level of engagement in the task, considering two Inclusion x Percentage models (100,66 and 100,33). None of the models showed significant group differences. But at pFWE<0.05 (cluster-forming p<0.001) for group average, both models showed clusters that survived peak-level correction (Table 1). 

The neuromorphometrics atlas from SPM12 revealed significant (p<0.05 FWE-corrected at peak level) areas intersecting with the areas including IOG, Calc, and Cun for Inclusion x Percentage (100,66) (Table 1 and Figure 2). We also found significant (p<0.05 FWE-corrected at peak level) intersecting areas, including IOG, LiG, and Calc, for Inclusion x Percentage (100,33) (Table 1 and Figure 3).

Correlation effects of childhood trauma

We assessed the influence of childhood trauma on the models (the main and interaction effects) related to whether those relationships differed between the controls and patients with BPD. Voxels survived at pFWE<0.05 corrected for peak level for the slope differences, and the average for all the models included ventral diencephalon (VD), POrG, MOrG, brain stem, ITG, FuG, and postcentral gyrus (PoG) (Table 2).

**Table 2:** Influence of childhood trauma on different models (F-contrast) ^1^Locations based on the two largest intersections for the peak level correction at pFWE<0.05 (cluster-forming p<0.001) based on the SPM neuromorphometrics atlas; SA: Slope average; SD: Slope difference; pFWE: peak-level family-wise error; SPM: Statistical Parametric Mapping; CTQ: Childhood Trauma Questionnaire

Slope	Metrics of CTQ	Cluster size	Peak F-statistic	Peak Coordinates (mm)			Cluster location^1^
				x	y	z	
Inclusion vs. Exclusion							
SA	Sexual abuse	44	40.18	8	-8	-16	Ventral Diencephalon—VD (49.1%), Unknown (31.7%)
100% Inclusion vs. 66% Inclusion							
SD	Emotional abuse	72	30.71	30	32	-6	Cerebral White Matter (71.4%), Posterior orbital gyrus—POrG (8.7%)
SD	Physical abuse	80	36.48	32	34	-6	Cerebral White Matter (72.5%), Posterior orbital gyrus—POrG (7.8%)
Inclusion × Percentage (100, 66)							
SD	Emotional neglect	25	34.25	-18	46	-10	Cerebral White Matter (76.9%), Medial orbital gyrus—MOrG (9.6%)
SD	Sexual abuse	39	34.21	8	10	-16	Ventral Diencephalon - VD (55.3%), Unknown (29.4%)
SA	Sexual abuse	42	35.65	8	-10	-16	Ventral Diencephalon - VD (55.3%), Unknown (29.4%)
SA	Sexual abuse	64	33.46	-14	-20	-18	Ventral Diencephalon - VD (41.5%), Brain Stem (17.3%)
Inclusion × Percentage (100, 33)							
SD	Sexual abuse	73	34.65	50	-54	-20	Inferior temporal gyrus—ITG (63.0%), Fusiform gyrus—FuG (15.1%)
SA	Sexual abuse	76	38.76	12	-34	74	Postcentral gyrus—PoG (44.1%), Unknown (20.5%)

## Discussion

In this study, 20 patients with BPD and 16 healthy controls played Cyberball, a virtual ball-tossing game, while the probabilities of getting the ball (0%, 33%, 66%, and 100%) were experimentally manipulated to induce an environment of social exclusion. The analysis focused on detecting the neural substrates of social exclusion and its association with childhood trauma in patients with BPD compared to healthy subjects. The findings of the study revealed brain areas with main and interaction effects depending on the models considered for the fMRI analysis.

Comparison with previous literature

There is a probable connection between disrupted emotion processing and other prominent features of BPD, such as impulsivity and interpersonal difficulties due to limbic hyperreactivity and decreased recruitment of the frontal brain regions [21]. Persistent sexual or physical abuse or emotional or physical neglect caused by childhood trauma alters the function of the limbic system and HPA axis, playing a significant role in cognitive processes involved in learning, long-term memory, and emotional processing in response to social exclusion [10]. We found significant activations in the brain areas in the fronto-limbic network, including regions involved in emotional processing and the insula (Table 1). Additionally, activations were also found in frontal brain regions engaged in regulatory control processes like SFG, MFG (Figure 1), and OFC, which are implicated in individuals with BPD, contributed by structural and functional changes in these networks [10].

Patients with BPD also exhibited increased grey matter (GM), mainly in the supplementary motor area extending to the right PCC and bilateral primary motor cortex, right MFG, and the bilateral cuneus extending to bilateral PCC. Decreased GM was identified in bilateral MTG, the right inferior frontal gyrus (IFG) extending to the right insula, the left hippocampus, and the left SFG extending to the left medial orbitofrontal cortex, suggesting that patients with BPD have significant GM abnormalities in the DMN and the fronto-limbic circuit [22], as depicted in our findings (Table 1).

Among the significant areas surviving pFWE correction, SPL (Table 1) contains Brodmann area (BA) 5 and 7, which play a particular role in visually guided visuomotor and observational processes, execution, reasoning, attention, and working memory [23] involved in the coherent tossing of balls within the experimental task. As a part of the insular cortex (BA 16), AIns generates predictions of future bodily states, computes the error by comparing the predicted signals with actual sensory signals, and integrates the body with the mind by minimizing the prediction error [24]. Also, AIns (Table 1) is implicated in patients with BPD, resulting in specific neuropsychiatric symptoms, such as difficulty in emotion regulation, depression, anger, and depressive rumination [25]. 

The IOG, LiG, Cun, and PCu areas observed in our study areas (Table 1, Figures 2, 3) are known to be part of the BA 19, which is primarily a visual association area with feature-extracting, shape recognition, attentional, and multimodal integrating functions, along with BA 18. Understandably, the visual processing and emotional responses to exclusion from the game involved during task performance led to these activations in the IOG in the participants. The areas of intersection found in our evaluation, MTG, FuG, and occipital fusiform gyrus (OFuG; Tables 1,2), comprising BA 37 and 38, are involved in cognitive processes for visual categorization, object naming, recognition memory, high-level semantic representation, and socio-emotional processing [26]. BA 37 and 38 also provide direct neural connections to the anterior orbital gyrus (AOrG) and orbitofrontal cortex (BA 10 and 11), which influence the decision-making process in the frontal lobe, interfering with the functionality for social and emotional processing [27]. Functional alterations in MTG and AIns (Table 1), besides the PHG, are also evident in higher traumatic events in childhood, affecting the network transmission efficiency of regions involved in cognitive and executive functions of the adult brain [8]. Individuals with BPD also show altered activations of the temporoparietal junction (TPJ), MFG, the amygdala, insula, and ACC modified by prior experiences of childhood neglect [9]. Out of these regions, we detected noticeable activations in MFG and the insula (Table 1 and Figure 1), despite a significant area regulating emotional processing, the amygdala, being missed in our findings.

Strengths and limitations

The study provides insights into the areas of significant activations in the brain related to the responses to social abandonment and traumatic childhood experiences in individuals with BPD. The fMRI analysis could not detect signals from the areas, including the amygdala, hippocampus, and ACC, as parts of the dysfunctional fronto-limbic system. In addition, our voxel-wise evaluation also missed areas in the ACC and medial PFC, consisting of the medial precentral area (PrCm), prelimbic cortex (PL), and infralimbic cortex (IL), that play a central role in mediating the interpersonal communication that is characteristic of BPD. The study also provided some interesting findings in terms of differences in anticipatory responses towards social exclusion based on the type of abuse or trauma experienced in childhood in BPDs. Yet, a detailed analysis of the signals differentiating the effects of different varieties of traumas (i.e., physical, emotional, and sexual) was not conducted, as they were considered beyond the scope of this study. In addition, the unequal sample size between the patients with BPD and the controls was also considered as a limitation, yet inevitable since images from four controls were discarded due to faulty fMRI during the scans.

Further studies

Further evaluation based on sex and subgroups of comorbidities of patients with BPD is necessary since the sex-based differences in symptoms suggest that male patients display more aggressiveness and impulsivity, and the female patients show an increased occurrence of affective instability, suicidal or self-harm behaviors, and unstable relationship(s) [28]. Besides, increased comorbidities of substance abuse and cluster A and B personality disorders observed in both genders show a greater likelihood of comorbid anxiety, eating, and mood disorders [28]. Additionally, future analysis considering the differentiation of signal changes across the hemispheres and the effects of medications on patients with BPD can enlighten our understanding of the development and treatment of BPD with further details.

## Conclusions

Findings of our study revealed activations in brain areas consistent with contemporary findings from fMRI studies conducted to evaluate social processing and the effects of childhood trauma in patients with BPD. We also found reduced sensitivity to changes in the social environment, suggesting increased anticipation of social abandonment in the individuals with BPD in comparison to the controls, attributed to the higher level of childhood adversity. However, our fMRI results were inclusive of the cortical changes caused by both social exclusion and childhood trauma, and further evaluation devised to explore the neural correlates for specific subtypes of childhood trauma and social exclusion could generate valuable insights into a better understanding of the etiology and psychopathology of BPD, contributing to an improved treatment strategy and quality care for patients with BPD. 
